# Iowa State Medical Society

**Published:** 1866-07

**Authors:** 


Adjourned *sine die.*

---

### ANNUAL MEETING OF THE IOWA STATE MEDICAL SOCIETY.

The Iowa State Medical Society met in the basement of the Second Baptist Church, Davenport, on Wednesday, the 9th of May, at 10 o'clock A. M., the attendance being fair and various parts of the State represented.

In the absence of the President, Dr. J. C. Hughes, of Keokuk, Dr. J. Williamson, of Ottumwa, Vice-President, took the Chair and called the meeting to order. Dr. J. W. H. Baker, of Davenport, appeared as Secretary.

The Rev. Mr. Watson, of the Baptist Church, was called upon and opened the proceedings with a fervent prayer.

In the absence of Dr. J. M. Witherwax, President of the Scott County Medical Society, Dr. Thos. J. Saunders received the members, addressing them as follows:

*Gentlemen of the Iowa State Medical Society:*

Again we give you cordial and hearty welcome to the city of Davenport. Since the organization of the Society, sixteen years ago, this is the third time we have been honored by your presence. The meeting of 1859, particularly, has a strong abiding place in the memory, invested as it was by an intensity of zeal and development of ability, promising as an eventual result of our associated capacity, great and permanent good. The disturbed state of the country for the past five years may have retarded somewhat our progress towards that liberal scheme of expansion which was intended to embrace and concentrate the Medical mind of Iowa. But your presence here to day is regarded as an harbinger of renewed interest in all that pertains to our noble calling—an interest, it is hoped, which, with the return of peace to the land, may go on from year to year constantly increasing.

May the time come when, instead of a limited number of delegates from those localities only which preponderate in population, our annual gatherings shall be composed of learned and experienced representatives from every county in the State. With Iowa's steady and rapid march as a commonwealth, the desire of every true votary of medical science within her borders should be to unfold and sustain our peculiar sphere of usefulness, in a manner fully equal to her constantly enhancing political greatness. If, in all our movements as a body, we are governed by reasonable prudence, animated by unflagging enthusiasm, and constantly recognize the importance of unity of action, then we have reason to believe the anticipated fruits of close organization will be made manifest, to some at least, who, from the very beginning, have been assiduously engaged in maintaining the dignity of the profession, as well as to those who now have control of the laboring oar.

With a vivid remembrance of the hospitality the physicians of Scott county have experienced in various parts of the State, we greet you on this occasion, and esteem it a privilege to have the opportunity of reciprocating the kindness which we have uniformly met when abroad.

The proceedings of the last annual meeting, held at Ottumwa, were then read by the Secretary and approved.

Dr. W. F. Peck, of Scott, offered the following, which was adopted:

*Resolved*, That Doctors Iles, Twining, Harrison, Blackburn, Hudson, Carpenter, French, Richardson and Rodman, be invited to seats in this body until they shall become members.

In accordance with the usual custom, the Constitution and By-Laws of the Society were then read by the Secretary.

The Censors of the Society all being absent, the following gentlemen were appointed to examine applicants for admission to membership, viz: Drs. Peck, Saunders, Watson, Whinnery, and Tomson. Soon after a report was made, and new members were unanimously elected by ballot (Drs. McCortney and Peck being tellers) as follows:

Dr. L. French, graduate of Berkshire Medical College, Massachusetts, Dec., 1853.

Dr. S. D. Richardson, graduate of Ohio Medical College, Cincinnati, O., March, 1839.

Dr. Thos. J. Iles, graduate of Transylvania University, Kentucky, March, 1838–9.

Dr. A. M. Carpenter, graduate of University, Louisville, Ky., March, 1854.

Dr. A. T. Hudson, graduate of Albany Medical College, N. Y., Feb., 1847.

Dr. J. C. Blackburn, graduate of Cincinnati College Med. and Surgery, Ohio, March, 1855–6.

Dr. A. A. Rodman, graduate of Keokuk Medical College, Iowa, March, 1864–5.

Dr. Watson, of Dubuque, offered the following, which was adopted:

WHEREAS, The regular annual meetings of this Society have been somewhat interrupted by the absence of many members of the profession in the service and from other causes, which has resulted in placing a large number upon the suspended list; therefore,

*Resolved*, That members suspended for the non-payment of dues be permitted to resume their standing in the Society at this meeting, by the payment of the annual fee for the present year.

On motion, the following committee on nominations for officers of the Society, was raised: Dr. Peck, of Scott; Dr. Carpenter, of Lee; Dr. J. C. Lay, of Dubuque; Dr. Rousseau, of Washington; Dr. Whinnery, of Lee; Dr. Carhart, of Linn; and Dr. Tomson, of Scott.

The presiding officer laid before the Society a communication from Mrs. Annie Wittenmyer, Matron of the Soldier's Orphans Home, extending an invitation to visit the institution, which was accepted, with the understanding that the time of said visit should hereafter be designated.

On motion of Dr. McCortney, the Society adjourned till 2 o'clock P. M.

### AFTERNOON SESSION.

At 2 o'clock P. M. the Society again met, Dr. Williamson taking the Chair.

The Secretary presented letters received from Dr. J.W. Robbins, of Manchester, and Dr. A. G. Field, of Des Moines, excusing themselves for non-attendance.

The Committee on Nomination asked for and obtained time.

Dr. Watson, Treasurer, made a report, which was accepted.

Dr. Gamble, from the Board of Censors, made a report in favor of the following persons for membership, who were subsequently balloted for and elected:

Dr. James Irwin, graduate of Geneva Medical College, N.Y., 1845.

Dr. George W. Carter, graduate of University of Michigan, 1853.

Dr. P. V. Clark, graduate of Keokuk Medical College, 1860.

Dr. W. A. Hosford, graduate of Castleton Medical College, Vt., 1846.

The regular call for reports of committees was then proceeded with.

Professor Carpenter read a highly interesting report prepared by J. C. Hughes, giving his experience in and advocacy of the bi-lateral operation for removal of urinary calculi. The statement was made that he had operated in all twenty-one times with only a single fatal termination; and that, four times successively, he had been called upon to perform upon the same person, a patient of 65 years of age. The report excited much interest and was received and referred to Committee on Publication. After being read, Drs. Peck, Baker, Williamson, Lay and Whinnery made each remarks, giving results of treatment of cases of urinary calculi coming under their notice.

Dr. Williamson laid before the Society a report prepared by Dr. Thrall, of Ottumwa. The reading of it was postponed until the evening session.

Dr. Baker read a report upon "New Remedies," which was received and referred to Committee on Publication. Upon its conclusion, a general disposition being manifested to give expression to a variety of views, the continuation of the subject was postponed until the evening session.

The reception of reports was now discontinued, and the Society proceeded to the consideration of miscellaneous business.

The Secretary presented a bill for expenses, which was referred to an auditing committee consisting of Drs. Whinnery and Iles.

The subject of appointing a time for visiting the Soldiers' Orphans Home agreeably to the invitation tendered this morning, was taken up, and after some discussion was laid upon the table for the present, a strong disposition being manifested to comply, if time would permit.

Dr. Saunders offered the following, which was adopted:

*Resolved*, That Professor A. M. Carpenter, of Keokuk, Dr. J. W. H. Baker, of Davenport, and Dr. Wm. Watson, of Dubuque, be appointed a committee for the purpose of presenting resolutions expressive of the sense of this body in relation to our late fellow associate, Professor D. L. McGugin, who, since our last meeting, has departed this life.

The Society then adjourned till 7½ o'clock this evening.

### EVENING SESSION.

The Society met at 7½ o'clock.

Dr. Williamson called Dr. Watson to the chair, and read a paper entitled "Hemato-Pathology, in its application to Diphtheria and Spotted Fever," prepared by Professor H. L. Thrall, of Ottumwa. The same was referred to the Committee on Publication.

The discussion of the subject of "New Remedies," then occupied the Society for the rest of the evening.

Then adjourned till 9 o'clock to-morrow (this) morning.

### SECOND DAY.

At 9 o'clock A. M., the Society was called to order, Dr. Williamson in the chair.

The Secretary being absent, Dr. W. F. Peck was elected to occupy the position temporarily.

Dr. Whinnery presented resolutions in relation to the manner of appointing Hospital Stewards at the State Prison, which, after some explanation, and with the mover's consent, was laid on the table for the present.

Dr. Gamble, from the Board of Censors, reported the following for membership, who were balloted for and elected:

Stephen M. Cobb, Maine Medical School, 1852.

Melville M. Shearer, Keokuk Medical College, 1864.

Dr. Peck made a report from the Committee on Nominations, which was received.

The amendment to the Constitution, offered by Dr. Baker at the meeting last year, "allowing the election of a permanent Secretary, and the location of the place of meeting at one point for the term of five or ten years next succeeding the season of 1866," was taken up and unanimously adopted.

Dr. Blackman offered the following resolution:

*Resolved*, That this Society shall hold its meetings annually for five years succeeding May 9th, 1866, at Davenport, Iowa.

The resolution, after some discussion, was adopted, having been amended so as to read "at Davenport, Iowa, or Des Moines, Iowa."

Prof. Carpenter made the following report:

The committee on resolutions expressive of the sense of this body relative to the death of Dr. D. L. McGugin, beg leave to report the following:

WHEREAS, Since the last meeting of the Iowa State Medical Society, by a dispensation of Divine Providence, our lamented associate, Dr. D. L. McGugin, has been removed from us by death, and summoned to his reward—therefore,

*Resolved*, That while we bow with submission to the stern decree, we mourn with sincere and heartfelt sorrow, the loss of our departed brother, and will ever cherish the memory of his unwavering child-like humility; his open, generous, manly virtues; his professional zeal, and his untiring Christian benevolence.

*Resolved*, That in his loss this Society is deprived of one of its most earnest co-laborers, whose life has been devoted with a singleness of purpose to the advancement and upbuilding of a profession honored among men.

*Resolved*, That we tender to the bereaved family of our deceased brother the heartfelt sympathies of this body, and that they be furnished with a copy of the proceedings.

A. M. CARPENTER,
*Chairman of Committee.*

The report having been read, the same was accepted and unanimously adopted.

Dr. Maxwell moved to take up the invitation to the Soldiers' Orphans Home, and that a committee of five be appointed to visit the institution and report.

Agreed to, and Drs. Cobb, Carpenter, Lay, Gamble and Hudson were appointed said committee.

The report of the Committee on Nominations was taken up, and the Society proceeded to the election of officers for the ensuing year, with the following result:

*President*—Dr. John W. H. Baker, of Scott.
*Vice-President*—Dr. James C. Lay, of Dubuque.
*Secretary*—Dr. Washington F. Peck, of Scott.
*Corresponding Secretary*—Dr. Abram M. Carpenter, of Lee.
*Treasurer*—Dr. M. B. Cochran, of Johnson.
*Censors*—Dr. Rousseau, of Washington; Dr. Schaffer, of Jefferson; Dr. Gamble, of Scott; Dr. Watson, of Dubuque, and Dr. Field, of Polk.

The recommendation of the Nominating Committee, that the next meeting of the Society be held at Davenport, was adopted.

The newly elected President was then conducted to the chair, and acknowledged the compliment of being chosen to the position in a brief and appropriate address.

The Society then proceeded to the election of delegates to the American Medical Association, to meet in Cincinnati next year, as follows: Dr. Thos. J. Saunders, Dr. A. M. Carpenter, Dr. Whinnery, Dr. Watson, Dr. Geo. W. Carter, Dr. Carhart, Dr. W. F. Rodman, Dr. Blackwell, Dr. Gamble, Dr. Hudson, Dr. Cobb, Dr. Lloyd, Dr. J. C. Lay, Dr. Williamson, and Dr. J. M. Schaffer, the delegates to have the power of substitution from members of the State Society.

The Society then adjourned till 2 o'clock P. M.

AFTERNOON SESSION.

At 2 o'clock P. M., the President, Dr. Baker, took the chair and called the meeting to order.

Drs. Gregg, Truesdale, and Plummer, of Rock Island Medical Society, being present, were invited to take seats in the meeting.

Dr. Whinnery presented some interesting cases in connection with Obstetrics, occurring in his practice, which were read and referred to the Committee on Publication.

Dr. Peck presented a paper upon a medical topic, as chairman of a committee raised for the purpose of investigating its history and treatment, and read the same to the Society. It was referred to the Committee on Publication.

Dr. John H. Rauch, former President of the Iowa State Medical Society, then delivered an eloquent address, which commanded the undivided attention of the members for more than an hour. Upon its conclusion, the thanks of the Society were tendered to Dr. Rauch and a copy requested for publication.

The following resolutions presented by Dr. Whinnery this morning, were taken up and adopted:

*Resolved*, In view of the fact that the Hospital at the Iowa Penitentiary is one of the best positions in the State for young men to study medicine, that the Warden be requested by this Society to give the preference to those who are about to enter upon the study of our profession for the Stewardship.

*Resolved*, That the institution owes this to the Medical profession, and that if the present Warden does not grant it agreeably to the desire of the attending physician, we respectfully request the Legislature at its next session to pass an act making it imperative to do so.

On motion, The thanks of the Society were unanimously tendered to the Rev. Mr. Watson and those having charge of the Baptist Church for the use of their Lecture-room during their stay.

Dr. Williamson offered the following, which was adopted:

*Resolved*, That all old Committees on diseases and subjects for report, are hereby discharged and that new ones be formed.

On motion of Dr. Watson, Drs. Gamble and Saunders were appointed to act in connection with the President as Committee on Publication.

Adjourned to meet at Dr. Iles' office at 7 o'clock P. M.

EVENING SESSION.

At 7 o'clock P. M., the Society met at the office of Dr. Iles, the President in the chair.

Various committees were appointed for the purpose of reporting at the next Annual Meeting.

Dr. Gamble, from the Board of Censors, reported for membership as follows, and a ballot resulted in their unanimous election:

Dr. Samuel Knox, Graduate of Penn'a Medical College, Pa., 1857.

Dr. James Guthrie, Graduate of Western Reserve College, Ohio, 1860.

The amount of matter to be published was left to the discretion of the Committee on Publication.

The Committee appointed to visit the Soldiers' Orphans Home made a report that greater attention to the sanitary condition of the vicinity would, in their opinion, be of advantage to the health of the inmates; also, that the entire control of the matter should be under the direction of the medical officer. Report adopted.

The Society then adjourned *sine die*.

---

